# A dopamine electrochemical sensor based on a platinum–silver graphene nanocomposite modified electrode†

**DOI:** 10.1039/c9ra11056a

**Published:** 2020-05-05

**Authors:** Nadzirah Sofia Anuar, Wan Jeffrey Basirun, Md. Shalauddin, Shamima Akhter

**Affiliations:** Department of Chemistry, Faculty of Science, University of Malaya Kuala Lumpur 50603 Malaysia sofiazira92@gmail.com jeff@um.edu.my +603 7967 4193 +603 7967 4082; Institute of Nanotechnology and Catalysis (NanoCat), University of Malaya Kuala Lumpur 50603 Malaysia

## Abstract

A platinum–silver graphene (Pt–Ag/Gr) nanocomposite modified electrode was fabricated for the electrochemical detection of dopamine (DA). Electrochemical studies of the Pt–Ag/Gr nanocomposite towards DA detection were performed by cyclic voltammetry (CV) and differential pulse voltammetry (DPV). The CV analysis showed that Pt–Ag/Gr/GCE had enhanced electrocatalytic activity towards DA oxidation due to the synergistic effects between the platinum–silver nanoparticles and graphene. The DPV results showed that the modified sensor demonstrated a linear concentration range between 0.1 and 60 μM with a limit of detection of 0.012 μM. The Pt–Ag/Gr/GCE presented satisfactory results for reproducibility, stability and selectivity. The prepared sensor also showed acceptable recoveries for a real sample study.

## Abstract 2

A platinum–silver graphene nanocomposite was synthesized and characterized. A nanocomposite modified electrode was fabricated in order to investigate the electrochemical detection of dopamine.

## Graphical abstract

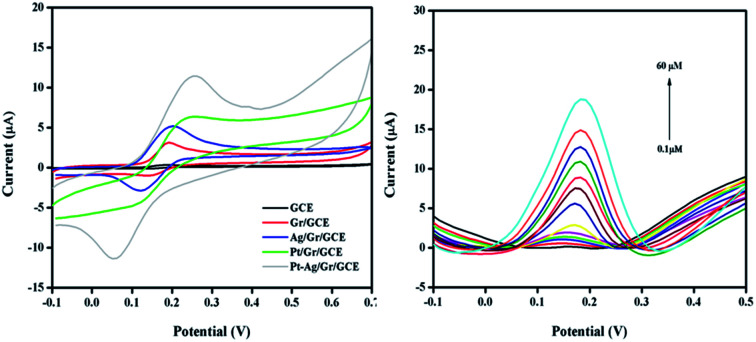

## 1. Introduction

Dopamine (DA), a neurochemical transmitter in the brain was discovered by Arvid Carlsson in 1957.^1^ Dopamine (DA) affects the cardiovascular, central nervous, endocrine and renal systems.^2^ DA also controls certain physiological conditions such as attention, learning, memory, movement, mood, behaviour and mental cognition.^3,4^ Imbalance in DA levels in the human brain may cause depression, addiction, schizophrenia and neurodegenerative diseases such as Alzheimer's and Parkinson's.^3,5^ Thus, the construction of a facile, selective and sensitive method for DA detection is necessary for monitoring DA levels in the human body. Several methods have been employed for the determination of DA such as chemiluminescence, chromatography, colorimetric, fluorescence and spectrophotometry.^6–8^ However, these conventional methods involve tedious sample pre-treatments, expensive and are time consuming.^9,10^ In recent years, electrochemical methods of detection have gathered enormous interest due to the simplicity, low cost, fast response time, low detection limit, high sensitivity and selectivity.^11,12^

Noble metal nanoparticles such as gold (Au), palladium (Pd), platinum (Pt) and silver (Ag) are broadly utilized as modified electrodes for electrochemical sensor applications.^13,14^ The superior conductivity and electrocatalytic activity of Pt makes it an attractive material for sensor applications.^15,16^ Since Pt is overpriced due to the low natural abundance,^17^ attempts have been proposed to minimize the use of Pt without a compromise in the electrocatalytic activity. The first approach is the introduction of a secondary metal, such as Ag, Cu, Fe and Ni, to form bimetallic nanoparticles. The bimetallic nanoparticles possess better electrochemical performance than the monometallic nanoparticles as a result of the synergistic effect between the two metals.^18^ Ag nanoparticles are recommended for the alloying with Pt due to their biocompatibility, electrochemical stability, abundance, low cost and sustainable electrocatalytic activity.^19^ Therefore, the coupling of Ag with Pt could lead to an enhancement in the electrocatalytic activity of the bimetallic nanoparticles. The second approach is to select a suitable support material for the distribution of bimetallic nanoparticles. Graphene, a monolayer of sp^2^ hybridized carbon atoms in a hexagonal framework,^20^ meets these requirements as a support material due to its amazing physical and chemical properties. Graphene offers a high surface area, excellent electrical conductivity, good chemical and thermal stability, and low production cost.^21^ Thus, the combination of graphene and bimetallic nanoparticles could improve the electrocatalytic activity of the nanocomposite.

Thus, a nanocomposite of platinum–silver bimetallic nanoparticles supported on graphene (Pt–Ag/Gr) was synthesized and characterized for morphological and structural properties. The electrochemical performance of the Pt–Ag/Gr/GCE towards the detection of DA was analysed using voltammetric techniques. From these results, the bimetallic Pt–Ag/Gr nanocomposite showed enhancement in the electrocatalytic activity towards DA detection compared to single metallic Pt/Gr and Ag/Gr nanocomposites.

## 2. Experimental methods

### 2.1. Materials

Dipotassium hydrogen phosphate (K_2_HPO_4_), potassium dihydrogen phosphate (KH_2_PO_4_) and potassium tetrachloroplatinate (K_2_PtCl_4_) were procured from Merck, while dopamine and silver nitrate (AgNO_3_) were acquired from Sigma Aldrich. Dopamine hydrochloride (DA) injection was supplied by University Malaya Medical Centre (UMMC). All reagents and chemicals were of high purity and used as received. The supporting electrolyte was 0.1 mol L^−1^ phosphate buffer solution (PBS) at pH 6.5. Desired volumes of K_2_HPO_4_ and KH_2_PO_4_ were mixed with Milli-Q water for the preparation of PBS, while the DA stock solution (0.01 mol L^−1^) was prepared by dilution in PBS.

### 2.2. Synthesis

#### 2.2.1. Synthesis of graphene

Graphene oxide (GO) was synthesized as the starting material for the preparation of graphene by a microwave-assisted method.^22^ First, graphite oxide was prepared from graphite following Hummers' method.^23^ Five milligrams of the prepared graphite oxide powder was sonicated in 25 mL distilled water for 40 min in an ultrasonic bath to produce a GO suspension. Then, the suspension was heated by a microwave system for 5 min. Subsequently, the graphene suspension was centrifuged before being placed in an oven at 60 °C for drying.

#### 2.2.2. Synthesis of Pt–Ag/Gr nanocomposite

The preparation of Pt–Ag/Gr was carried out by a chemical method as reported earlier.^24^ First, an equal amount of 0.05 mol L^−1^ AgNO_3_ and 0.05 mol L^−1^ K_2_PtCl_4_ aqueous solutions were mixed with a 10 mg mL^−1^ graphene suspension. Next, 0.1 mol L^−1^ sodium hydroxide solution was used to adjust the pH of the mixture to 8.0. The resulting mixture was stirred and heated at 60 °C for 35 min. This was followed by the addition of 0.01 mL hydrazine hydrate to the mixture while stirring and heating for an additional 30 min where the Ag and Pt nanoparticles were deposited onto the graphene surface to produce Pt–Ag/Gr. Subsequently, the Pt–Ag/Gr suspension was centrifuged before drying in an oven at 60 °C. The single metal Ag/Gr and Pt/Gr nanocomposites were also synthesized using the same procedure for comparisons.

#### 2.2.3. Preparation of the Pt–Ag/Gr modified electrode

For the preparation of the modified electrode, a suspension was prepared by dispersing 1.0 mg Pt–Ag/Gr nanocomposite in 1.0 mL Milli-Q water. Then, 10 μL of Nafion solution was added into the suspension, followed by ultrasonication for an hour to produce a homogenous dispersion. Next, 10 μL of the suspension was pipetted using a micropipette and drop-casted onto the glassy carbon electrode (GCE) surface. Finally, the prepared electrode was dried overnight at room temperature. For comparisons, Ag/Gr/GCE, Pt/Gr/GCE and Gr/GCE were also prepared using the same procedures.

### 2.3. Characterizations

The crystal structures of the nanocomposite were evaluated by a PANalytical EMPYREAN X-ray diffractometer with Cu Kα radiation (*λ* = 1.541 Å). The surface morphology of the samples was analysed using JEOL JSM-7600F field emission scanning electron microscopy (FESEM), while the particle size was analysed by Hitachi HT-7700 transmission electron microscopy (TEM), attached with an EDAX TEAM energy dispersive X-ray (EDX) spectrometer. Raman spectroscopy was performed using a Renishaw inVia Raman microscope with a laser source of 532 nm excitation wavelength. The X-ray photoelectron spectroscopy (XPS) instrument was a ULVAC-PHI Quantera II X-ray photoelectron spectrometer with an Al-Kα X-ray source. Fourier transformed infrared (FTIR) spectra were obtained using a PerkinElmer Spectrum 400 FTIR spectrometer.

### 2.4. Electrochemical study

Electrochemical experiments such as cyclic voltammetry (CV), differential pulse voltammetry (DPV) and electrochemical impedance spectroscopy (EIS) were performed by an Autolab potentiostat/galvanostat model PGSTAT302 (EcoChemie, Netherlands). All electrochemical experiments were performed using a three-electrode system consisting of Pt–Ag/Gr/GCE as the working electrode, saturated calomel electrode (SCE) as the reference electrode and a platinum wire as the counter electrode. All experiments were carried out at room temperature.

## 3. Results and discussions

### 3.1. Characterization of Pt–Ag/Gr

**Fig. 1:**
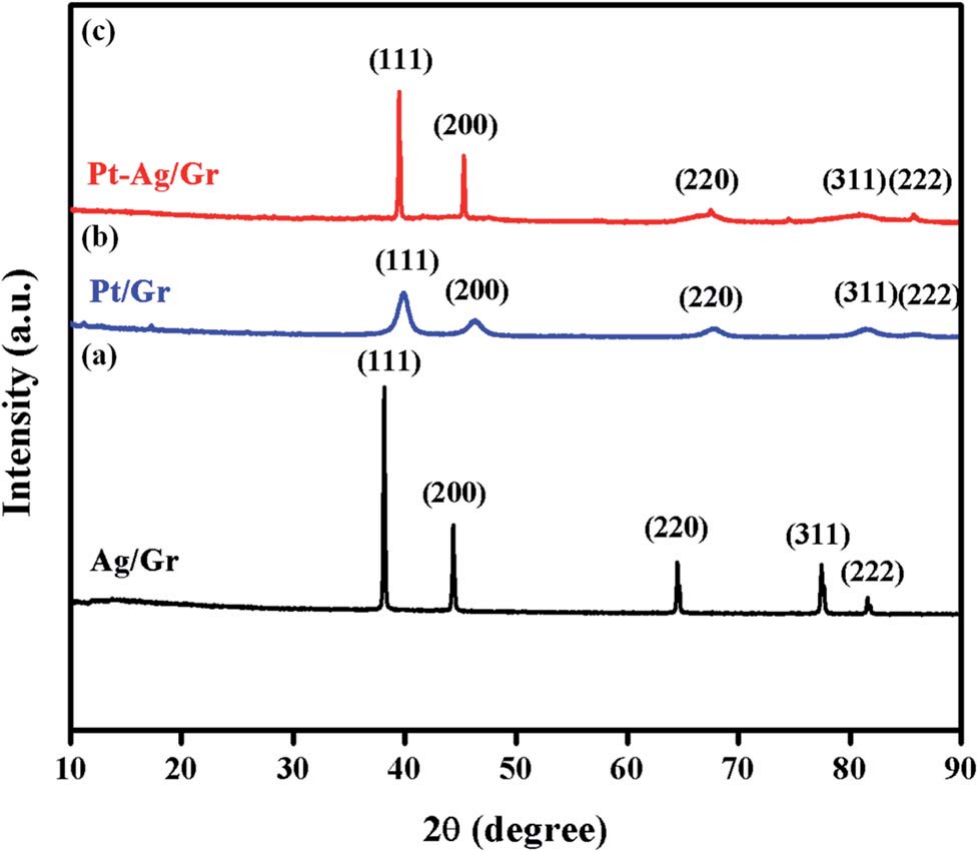
The XRD pattern of (a) Ag/Gr (b) Pt/Gr and (c) Pt–Ag/Gr nanocomposites.

XRD was performed to identify the crystal structures of the Ag/Gr, Pt/Gr and Pt–Ag/Gr nanocomposites and to confirm the formation of bimetallic Pt–Ag. Fig. 1a shows five diffraction peaks of Ag at 2*θ* values of 38.17°, 44.35°, 64.66°, 77.66° and 81.82° which correspond to the (111), (200), (220), (311) and (222) crystal planes, respectively. Additionally, the XRD pattern of the Pt/Gr nanocomposite (Fig. 1b) demonstrates characteristic peaks at 2*θ* values of 39.87°, 46.26°, 67.68°, 81.43° and 86.12°, which are assigned to the (111), (200), (220), (311) and (222) crystal planes, respectively. According to the results, both Ag and Pt have a face-centred cubic (fcc) crystal structure (Ref. codes: 00-065-2871 and 01-001-1194). The diffraction peaks of Pt–Ag/Gr are present at 2*θ* values of 39.52°, 45.32°, 66.28°, 81.01° and 85.70° (Fig. 1c) and are situated between the values of pure Ag (Ref. code: 00-065-2871) and Pt (Ref. code: 01-001-1194), which confirms the formation of bimetallic Pt–Ag.^25^ The mean particle size of Pt–Ag is 6.3 nm from the Scherrer equation, while the *d*-spacing of Pt–Ag is 0.228 nm. The *d*-spacing value is between that of Ag (0.236 nm) and Pt (0.226 nm).

**Fig. 2:**
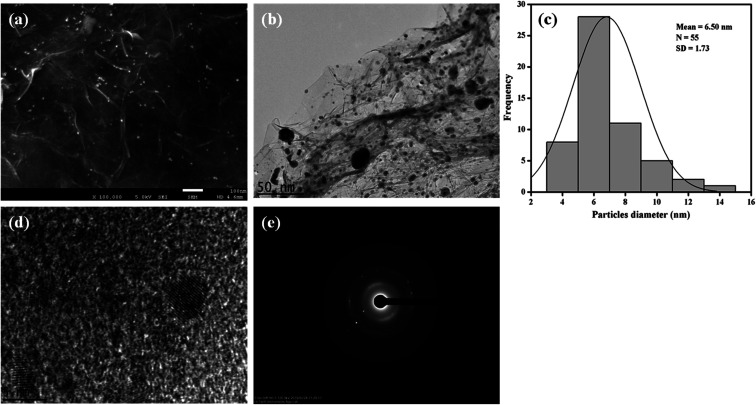
(a) FESEM image of Pt–Ag/Gr. (b) TEM image of Pt–Ag/Gr. (c) The particle size distribution histogram of Pt–Ag. (d) HRTEM image of Pt–Ag/Gr. (e) SAED pattern of Pt–Ag.

The morphological features of the as-synthesized Pt–Ag/Gr nanocomposite were analysed by FESEM and TEM imaging. The FESEM image in Fig. 2a illustrates the deposition of Pt–Ag nanoparticles on the wrinkled sheet structures of graphene. TEM analysis was performed to further validate the FESEM characterization. Fig. 2b presents the TEM image of the Pt–Ag/Gr nanocomposite. The TEM image shows that the graphene sheets are decorated with small spherical Ag and Pt nanoparticles. The mean nanoparticle size (*n* = 55, Fig. 2c) of Pt–Ag is approximately 6.50 nm, in the range of 4.0–14.0 nm. The small size of the nanoparticles and narrow particle size distribution suggest that the graphene support plays an essential role in the nucleation of the nanoparticles. The presence of carbon, oxygen, platinum and silver in the Pt–Ag/Gr nanocomposite was detected by EDX as shown in Fig. S1a.† EDX elemental mapping was used to investigate the distribution of Pt and Ag nanoparticles on the graphene surface. Fig. S1b† illustrates the elemental mapping images of carbon, oxygen, silver and platinum. The EDX elemental mapping (Fig. S1b†) shows the presence of bimetallic Pt–Ag nanoparticles on the graphene surface. Fig. 2d presents a higher magnification TEM image of the Pt–Ag lattice fringes. The lattice fringe spacing is approximately 0.230 nm, attributed to the (111) crystal plane of face-centred cubic Pt–Ag. The result is similar to the *d*-spacing value obtained from the XRD analysis. Fig. 2e presents the selected area electron diffraction (SAED) image with a definite ring, which reveals the presence of a crystalline material.^21^

**Fig. 3:**
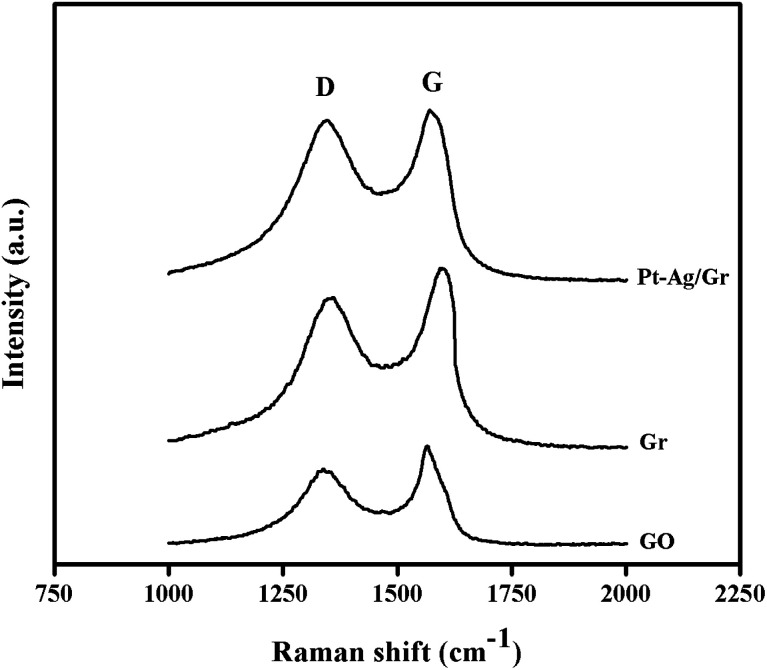
Raman spectra of GO, Gr and Pt–Ag/Gr.

The structural characteristics of carbon compounds especially graphene based materials can be investigated by Raman spectroscopy. The Raman spectra of GO, graphene and the Pt–Ag/Gr nanocomposite are shown in Fig. 3. Two distinctive bands at approximately 1360 cm^−1^ and 1580 cm^−1^ were identified in all the samples, which are attributed to the D and G bands, respectively. The presence of the D band is related to the defects and disorder in the graphene structure,^26^ whereas the G band originates from the E_2g_ vibrations of the sp^2^ carbon atoms.^27,28^ The intensity ratio of the D band and G band (*I*_D_/*I*_G_) represents the amount of disorder in carbon compounds.^29^ The *I*_D_/*I*_G_ of GO and graphene is 0.808 and 0.869, respectively, which suggests an increase in the defects in graphene synthesized from the reduction of GO with microwave irradiation. Furthermore, the *I*_D_/*I*_G_ ratio of the Pt–Ag/Gr nanocomposite (0.950) shows an increase in the defect density which confirms structural distortion of graphene due to the incorporation of Pt–Ag nanoparticles.

The chemical state and elemental composition of the prepared Pt–Ag/Gr nanocomposite were characterized by XPS. Fig. S2(a–d)† shows the survey scan spectrum of Pt–Ag/Gr with the deconvolution of the C 1s, Ag 3d and Pt 4f core level spectra. Three predominant peaks are present in the survey scan spectrum of Pt–Ag/Gr (Fig. S2a†) which are attributed to the C 1s, Ag 3d and Pt 4f levels. The C 1s core level spectra of Pt–Ag/Gr in Fig. S2b† shows three distinct peaks located at binding energies of 284.63 eV, 285.68 eV and 287.31 eV, which are ascribed to the C=C, C–O and C=O groups, respectively. As shown in Fig. S2c,† the deconvolution of the Ag 3d spectrum shows a doublet of Ag 3d_5/2_ and Ag 3d_3/2_ due to spin–orbit splitting which is located at 368.24 eV and 374.25 eV, respectively. Furthermore, the Pt 4f_7/2_ and Pt 4f_5/2_ peaks can be observed in the high-resolution Pt 4f spectra of the Pt–Ag/Gr nanocomposite, as presented in Fig. S2d.† Each peak of Pt 4f_7/2_ and Pt 4f_5/2_ can be deconvoluted into three different oxidation states, namely the Pt^0^, Pt^2+^ and Pt^4+^. Pt^0^ 4f_7/2_ and Pt^0^ 4f_5/2_ and are centred at binding energies of 71.14 eV and 74.69 eV, respectively. The two distinct peaks at 72.56 eV and 76.37 eV are characteristic of the Pt^2+^ 4f_7/2_ and Pt^2+^ 4f_5/2_ levels, respectively, whereas the peaks at 74.21 eV and 78.24 eV are assigned to the Pt^4+^ 4f_7/2_ and Pt^4+^ 4f_5/2_, respectively. The XPS results confirmed that both Ag NPs and Pt NPs are present on the graphene surface, thus the Pt–Ag/Gr nanocomposite was successfully synthesized.

**Fig. 4:**
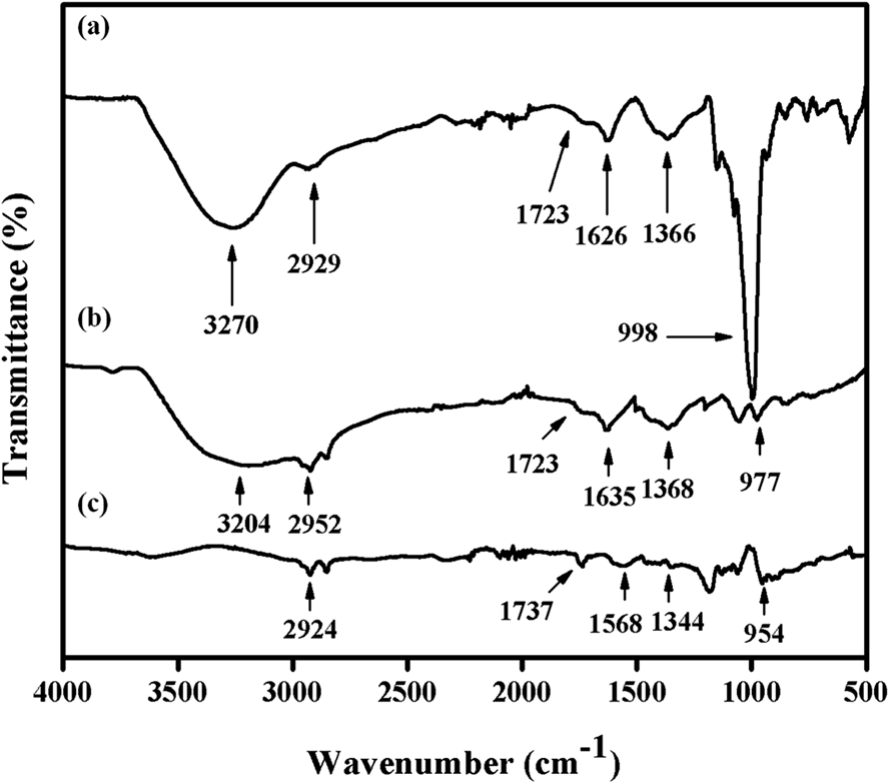
FTIR spectra of (a) GO, (b) Gr and (c) Pt–Ag/Gr.

Fig. 4 presents the FTIR spectra of GO, Gr and Pt–Ag/Gr in the range of 500–4000 cm^−1^ to investigate the functional groups.^30^ A broad band at 3270 cm^−1^ in the FTIR spectrum of GO (Fig. 4a) is assigned to the O–H stretching vibration of the GO sheets.^31^ The characteristic peaks at 2929, 1723, 1626, 1366 and 998 cm^−1^ correspond to the stretching vibrations of C–H, C=O, C=C, C–O–H and C–O, respectively.^19,25^ These results confirm that GO consists of oxygen functional groups.^6,31^ The peak intensities of the oxygen functional groups were diminished in Gr (Fig. 4b). In addition, some of the peaks were absent in Pt–Ag/Gr (Fig. 4c) which is likely due to the presence of Pt–Ag nanoparticles on the graphene surface.^5,32^ Thus, these results confirm that the oxygen functional groups were reduced in Pt–Ag/Gr.

### 3.2. Electrochemical impedance spectroscopy (EIS) studies

The interfacial electron transfer resistance (*R*_et_) of the modified electrodes was studied by EIS.^33^ Fig. S3† presents the Nyquist plots of the modified electrodes in a 5.0 mM Fe [(CN)_6_]^3−/4−^ redox couple in 0.1 M KCl between 100 kHz and 100 mHz. The Nyquist plots shows a semicircle at low frequency where the diameter corresponds to the interfacial electron transfer resistance (*R*_et_) of the modified electrodes. The low-frequency region shows a straight line 45° (Warburg element) to the horizontal axis, is due to a diffusion-limited process. The bare GCE shows the largest semicircle diameter which represents the largest electron transfer resistance. The semicircle diameter is considerably decreased when graphene is immobilized on the surface of the GCE. This is due to the high electrical conductivity of graphene, which decreases the impedance of the GCE. In addition, the semicircle diameter decreases progressively from Gr/GCE to Ag/Gr/GCE, Pt/Gr/GCE and Pt–Ag/Gr/GCE with the introduction of Ag NPs and Pt NPs on the graphene surface. This demonstrates the decreasing trend of the electron transfer resistance due to the deposition of conductive metal nanoparticles. The *R*_et_ values of GCE, Gr/GCE, Ag/Gr/GCE, Pt/Gr/GCE and Pt–Ag/Gr/GCE were obtained by fitting and simulation of the experimental impedance data with a suitable equivalent circuit. The *R*_et_ values decrease in the order of GCE (824 Ω) > Gr/GCE (533 Ω) > Ag/Gr/GCE (130 Ω) > Pt/Gr/GCE (116 Ω) > Pt–Ag/Gr/GCE (69 Ω). The *R*_et_ values of Ag/Gr/GCE and Pt/Gr/GCE are lower than those of Gr/GCE, implying that the presence of conductive metal nanoparticles facilitates the electron transfer process. Furthermore, Pt–Ag/Gr/GCE shows the lowest *R*_et_ values due to the synergistic effect of the bimetallic Pt–Ag nanoparticles and graphene which enhances the electrical conductivity and decreases the interfacial resistance. For this reason, Pt–Ag/Gr/GCE was utilized for the development of the electrochemical sensor.

### 3.3. Optimization of experimental conditions

#### 3.3.1. Effect of pH

The pH of the electrolyte significantly affects the electrochemical performance of DA at Pt–Ag/Gr/GCE. The cyclic voltammetry of DA were performed at different pH in 0.1 mol L^−1^ PBS between pH 5.5 and 7.5 in the presence of 25 μM DA. From Fig. S4a,† it can be seen that the peak potential for DA oxidation becomes less positive as the pH increases from 5.5 to 7.5, which confirms the involvement of protons in the electrochemical oxidation of DA.^34^ Furthermore, good linearity was achieved between the oxidation peak potentials and the pH values, as presented in Fig. S4b,† with a linear regression equation of *E*_pa_ (V) = −0.064pH + 0.681. From this equation, the obtained slope of 0.064 V pH^−1^ is close to the theoretical value from the Nernst equation (0.059 V pH^−1^) at standard temperature.^35^ This confirms that the electrochemical oxidation of DA involves an equal number of protons and electrons.^36^ The plot in Fig. S4c† illustrates that the DA oxidation peak current increases with the pH value from 5.5 to 6.5 but declines beyond pH 6.5. Therefore, pH 6.5 was chosen as the ideal pH for the electrochemical detection of DA in the subsequent experiments due to the highest DA oxidation peak current.

#### 3.3.2. Effect of scan rate

The influence of the scan rate on the redox peak currents of DA oxidation with Pt–Ag/Gr/GCE was studied by cyclic voltammetry. The cyclic voltammetric measurements were performed with the Pt–Ag/Gr/GCE at several scan rates from 5 to 200 mV s^−1^ in 0.1 mol L^−1^ PBS containing 25 μM DA as shown in Fig. S5a.† The redox peak current of DA increases with the scan rate, and a linear correlation between the parameters was observed. The regression equations shown in Fig. S5b† can be expressed as *I*_pa_ (μA) = 0.154*ν* + 0.676 (*R*^2^ = 0.988) and *I*_pc_ (μA) = −0.191*ν* − 7.55 (*R*^2^ = 0.971), which confirm that the electrochemical process is a surface adsorption controlled reaction.^37^ Furthermore, when the scan rate increases, the oxidation and reduction peak potentials are slightly shifted to positive and negative regions, respectively, which is associated with a slower electron transfer process at faster scan rates. Fig. S5c† demonstrates the linear relationship of the redox peak potential and the logarithm of the scan rate in the range of 5 to 75 mV s^−1^, with regression equations of *E*_pa_ = 0.0786 log *ν* + 0.184 (*R*^2^ = 0.962) and *E*_pc_ = −0.0564 log *ν* + 0.104 (*R*^2^ = 0.949). Based on the Laviron equation, the slopes of the two lines are 2.303*RT*/(1 − *α*)*nF* and −2.303*RT*/*αnF*. From the calculation, the electron transfer coefficient (*α*) and the number of electrons involved in the reaction are 0.58 and 1.8 ≈ 2, respectively. Therefore, two electrons are required in the electrochemical redox reaction of DA.

### 3.4. Electrochemical behaviour of dopamine on Pt–Ag/Gr/GCE

**Fig. 5:**
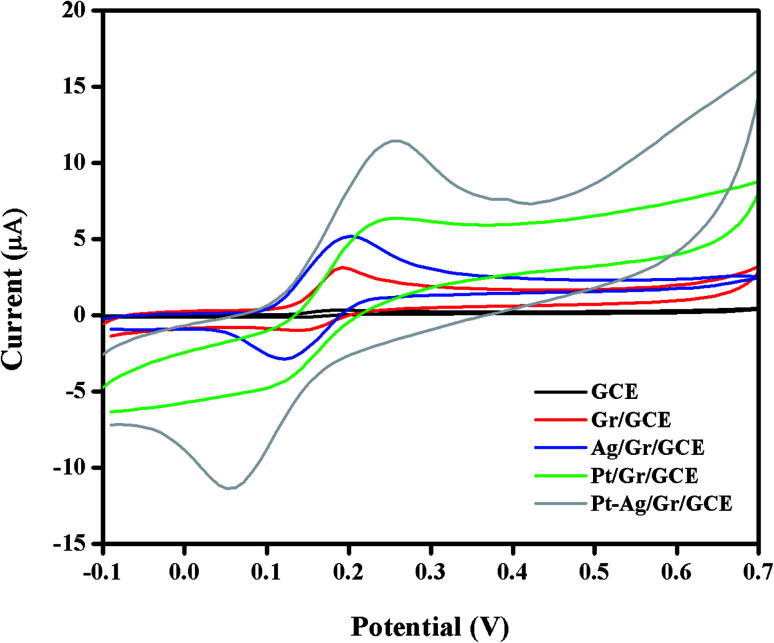
Cyclic voltammograms of 25 μM DA in 0.1 mol L^−1^ PBS (pH 6.5) at a scan rate of 20 mV s^−1^ on bare GCE, Gr/GCE, Ag/Gr/GCE, Pt/Gr/GCE and Pt–Ag/Gr/GCE.

The electrochemical performance of DA at the GCE, Gr/GCE, Ag/Gr/GCE, Pt/Gr/GCE and Pt–Ag/Gr/GCE was investigated by cyclic voltammetry in 0.1 mol L^−1^ PBS (pH 6.5) containing 25 μM DA at 20 mV s^−1^. In Fig. 5, only the oxidation peak current of DA was observed at the GCE due to the sluggish electrochemical reaction of DA on the GCE surface. In contrast, the Gr/GCE exhibited a pair of small redox peak currents. The DA oxidation peak current of the Gr/GCE increases slightly compared to the GCE due to the high surface area and high electron conductivity of graphene, which provides more active sites for the electrochemical reaction.^38^ Moreover, the presence of metal nanoparticles on the graphene surface facilitates the electron transfer reaction during the electrochemical detection of DA. The cyclic voltammograms of Ag/Gr/GCE, Pt/Gr/GCE and Pt–Ag/Gr/GCE show clear DA oxidation peak currents. The Pt–Ag/Gr/GCE shows the highest DA oxidation peak current compared to the Ag/Gr/GCE and Pt/Gr/GCE, confirming the outstanding electrocatalytic activity of Pt–Ag/Gr towards DA oxidation. The enhancement in the peak current is ascribed to the synergistic effects between the Pt–Ag bimetallic nanoparticles and graphene. The unique combination of both Pt and Ag provides excellent electrocatalytic performance and superior electronic conductivity compared to single metal Pt and Ag nanoparticles. Furthermore, the incorporation of Pt and Ag bimetallic nanoparticles on the high surface area of graphene contributes to more electroactive sites for efficient electron transfer between the modified electrode and analyte.^39^ Thus the Pt–Ag/Gr nanocomposite modified electrode is recommended for the electrochemical detection of DA due to the superior conductivity, excellent electrocatalytic activity and high surface area.

### 3.5. Analytical application of the sensor

**Fig. 6:**
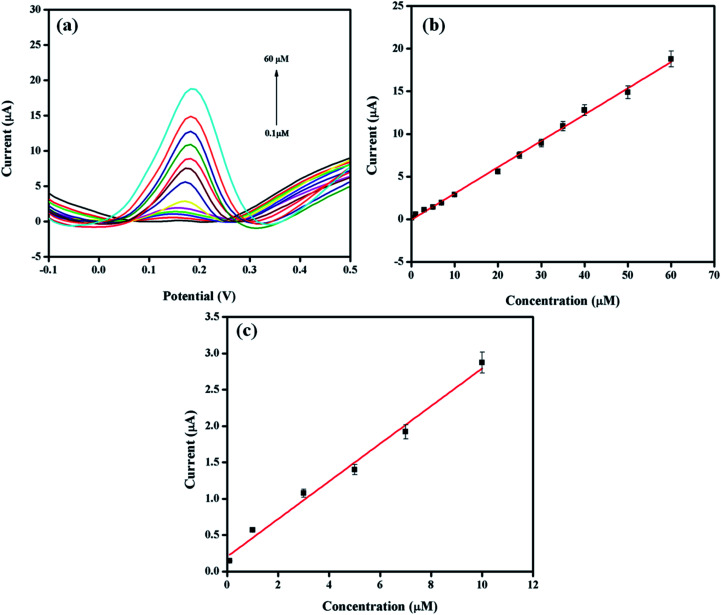
(a) Differential pulse voltammograms with various DA concentrations (0.1, 1, 3, 5, 7 10, 20, 25, 30, 35, 40, 50, 60 μM) at Pt–Ag/Gr/GCE in 0.1 mol L^−1^ PBS at pH 6.5. (b) Relationship between peak current and DA concentration. (c) The calibration curve in low concentration range.

The differential pulse voltammetry (DPV) method was selected for the quantitative analysis of DA on Pt–Ag/Gr/GCE due to the better sensitivity and lower detection limit than CV.^40^ The DPV responses of the sensor with various concentrations of DA in 0.1 mol L^−1^ PBS are presented in Fig. 6a. Based on the DPV results, the DA oxidation peak current increases with the DA concentration, from 0.1 to 60 μM. Fig. 6b demonstrates the excellent linearity of the calibration curve between the oxidation peak current and DA concentration, with a linear equation *I*_pa_ (μA) = 0.309*C* (μM) − 0.0699 (*R*^2^ = 0.997). The detection limit of DA is 0.012 μM based on a signal-to-noise ratio (S/N) of 3. The performance of Pt–Ag/Gr is compared with other modified electrodes for DA detection, as summarized in Table 1. The detection limit of Pt–Ag/Gr/GCE is comparable to β-CD/rGO and PA/GO and is even better than Au–Cu_2_O/rGO, N-rGO, Au/PDDA/GNS, PdAu/rGO, AG-NA, GNP/FTO, PGE, NGF and PANI/rGO. No perceptible differences were observed between the linear ranges of the present sensor with N-rGO, PdAu/rGO, NGF, PANI/rGO and β-CD/rGO. Thus, it can be concluded that the high sensitivity of Pt–Ag/Gr/GCE towards the electrochemical determination of DA is due to the synergistic effects of bimetallic Pt–Ag NPs and graphene.

**Table 1:** Comparison of analytical performance on different electrode materials for DA detection by DPV method

Electrode materials	Linear range (μM)	Detection limit (μM)	References
Au–Cu_2_O/rGO	10–90	3.9	38
N-rGO	3–70	1.5	7
Au/PDDA/GNS	2–28	1.0	41
PdAu/rGO	1.25–73.75	0.75	42
AG-NA	0.5–35	0.33	2
GNP/FTO	30–100	0.22	35
PGE	0.2–8	0.20	43
NGF	0.1–80	0.030	44
PANI-rGO	0.05–60	0.024	6
β-CD/rGO	0.05–50	0.017	36
PA/GO	0.05–10	0.016	45
Pt–Ag/Gr	0.1–60	0.012	[This work]

### 3.6. Stability and reproducibility of Pt–Ag/Gr/GCE

The stability and reproducibility of the Pt–Ag/Gr were tested to determine the efficiency of the sensor. For stability, the DA oxidation peak current was measured after storage of Pt–Ag/Gr/GCE in PBS for two weeks in a refrigerator. The electrochemical response of DA was reduced to approximately 11.8% of the initial current response value. Five different Pt–Ag/Gr/GCEs were prepared individually by the same method to investigate the reproducibility of the sensor towards DA detection. From the analysis, the relative standard deviation (RSD) of the DA oxidation peak current is estimated as 3.01%. These results suggest an acceptable storage stability and reproducibility of the prepared electrode.

### 3.7. Selectivity of Pt–Ag/Gr/GCE

The selectivity of a sensor towards a target analyte is one of the most essential characteristics of a high-performance sensor due to the presence of interfering compounds that also oxidize in the same potential range.^8^ The selective determination of DA in the presence of biological compounds such as ascorbic acid (AA), *p*-aminophenol (PAP), acetaminophen (AC) and uric acid (UA) was performed using DPV by varying the concentration of DA in a solution with a constant concentration of the interfering compounds. As shown in Fig. S6a,† the peak currents of AA, PAP, DA, AC and UA are separated from each other. Moreover, a linear relationship is observed between the DA peak current and the concentration, with the linear equation of *I*_pa_ (μA) = 0.292*C* (μM) + 14.59 as illustrated in Fig. S6b.† The sensitivity of the Pt–Ag/Gr modified electrode in the presence of interferences (0.292 A M^−1^) decreases only approximately 5.5% from the sensitivity of Pt–Ag/Gr/GCE in the absence of interference (0.309 A M^−1^). This implies that the presence of AA, PAP, AC and UA in the same mixture has a negligible effect on the detection of DA. Thus, Pt–Ag/Gr/GCE possesses acceptable selectivity for the detection of DA in the presence of common interferences.

### 3.8. Real sample analysis

Dopamine hydrochloride injection (40 mg mL^−1^) was used for the determination of DA at the Pt–Ag/Gr modified electrode in real sample analysis. The dopamine hydrochloride injection was diluted with 0.1 mol L^−1^ PBS (pH 6.5) for a suitable concentration range in the determination of DA. Several concentrations of dopamine hydrochloride injection were prepared, and the current response was examined using the DPV method under the same experimental conditions as before. From the results summarized in Table 2, the obtained recoveries are between 91.4–99.0%. Therefore, the fabricated sensor shows acceptable reliability for DA determination in real samples.

**Table 2:** DA determination in dopamine hydrochloride injection using Pt–Ag/Gr/GCE

Content (μM)	Detected (μM)	Recovery (%)	RSD (%)
3.00	2.74	91.4	2.94
8.00	7.92	99.0	1.25
10.0	9.56	95.6	0.58
12.0	11.9	98.8	1.76

## 4. Conclusions

In summary, a GCE surface was modified with Pt–Ag/Gr nanocomposite for the electrochemical determination of DA. From the results, the combination of graphene and Pt–Ag nanoparticles greatly improved the oxidation peak current of DA. The outstanding electrocatalytic activity of Pt–Ag/Gr towards DA was due to the synergistic effects of the high surface area of graphene and superior electronic conductivity of the Pt–Ag nanoparticles. The obtained Pt–Ag/Gr modified GCE had acceptable sensitivity, selectivity, stability, reproducibility and a low detection limit. With this remarkable performance, the prepared sensor was tested for the determination of DA in real samples with satisfying results.

## Conflicts of interest

There are no conflicts of interest to declare.

## Supplementary Material

RA-010-C9RA11056A-s001
