# Supplementary material for: A dopamine electrochemical sensor based on a platinum–silver graphene nanocomposite modified electrode
Source: RSC Adv. 2020 May 5;10(29):17336–44. doi: 10.1039/c9ra11056a (PMC9053441; doi:10.1039/c9ra11056a)
Supplement: RA-010-C9RA11056A-s001 [file RA-010-C9RA11056A-s001.pdf]

Electronic Supplementary Material (ESI) for RSC Advances.
This journal is © The Royal Society of Chemistry 2020

Supporting Information

Dopamine electrochemical sensor based on a platinum-silver graphene nanocomposite modified electrode - Nadzirah Sofia Anuar, Md. Shalauddin, Shamima Akhter, Wan Jeffrey Basirun

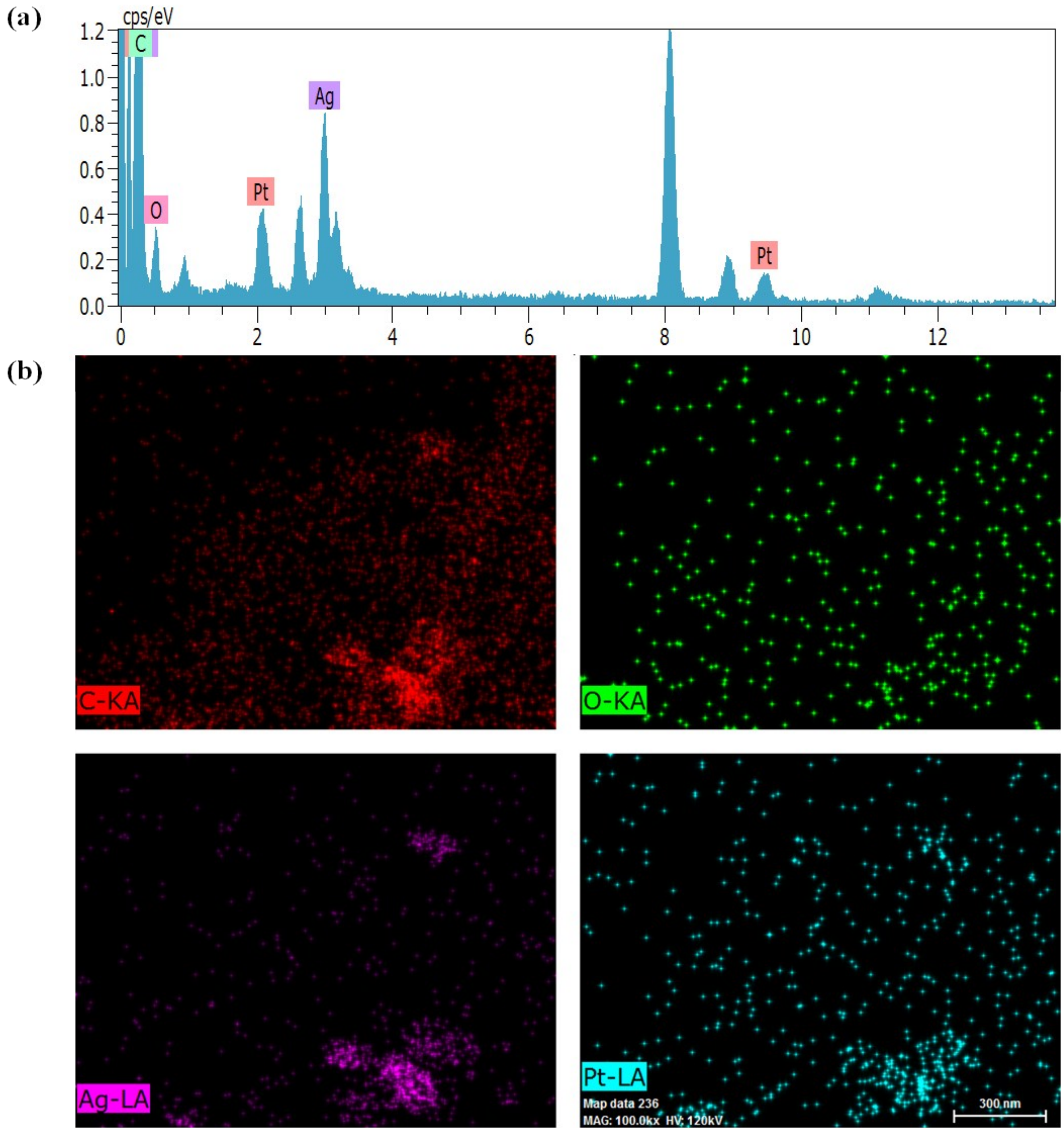

Fig. S1. (a) EDX spectra of Pt-Ag/Gr. (b) Elemental mapping images of carbon, oxygen, silver and platinum.

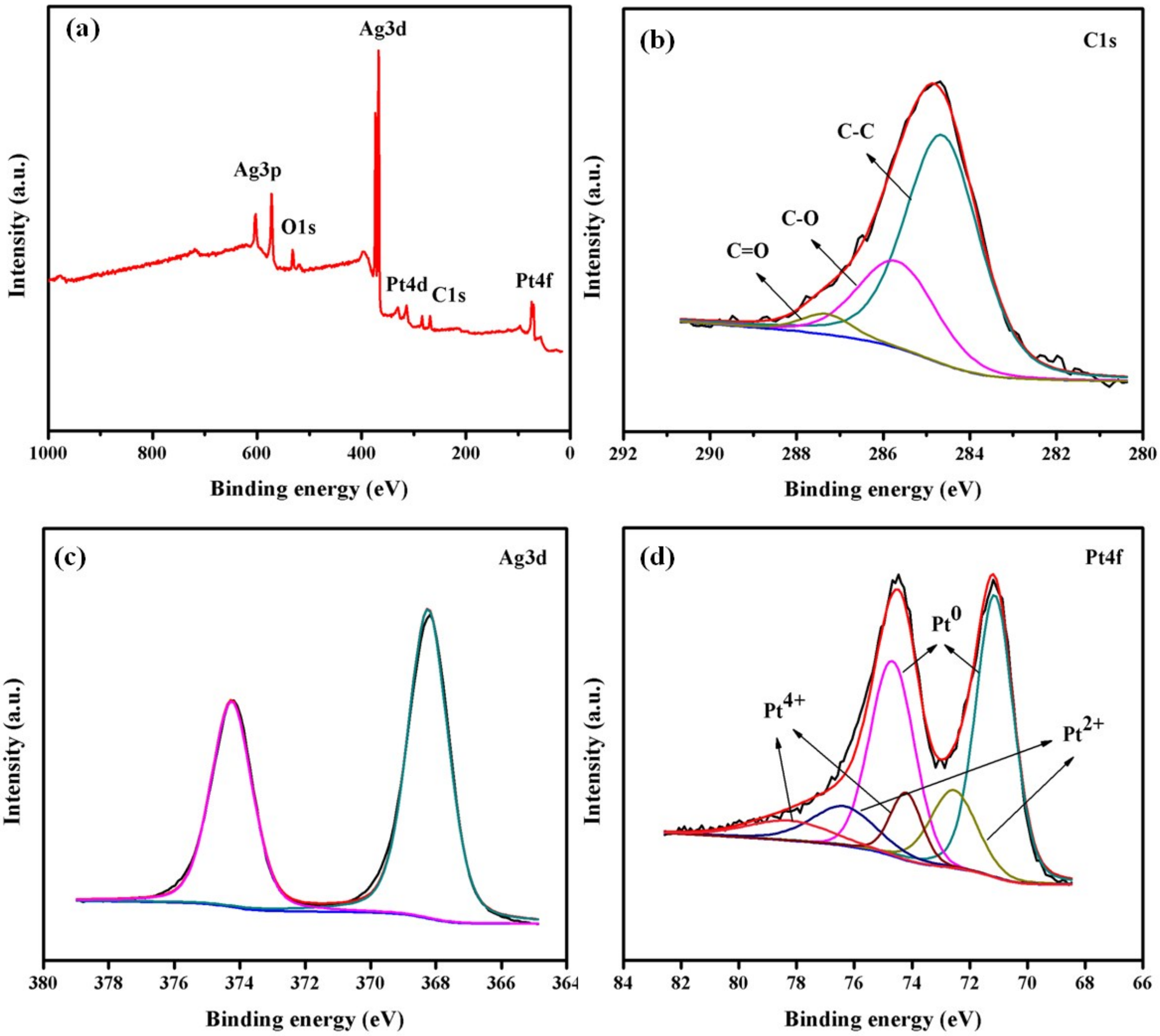

Fig. S2. XPS spectra of Pt-Ag/Gr nanocomposite: (a) Survey scan. High-resolution of (b) C 1s, (c) Ag 3d and (d) Pt 4f.

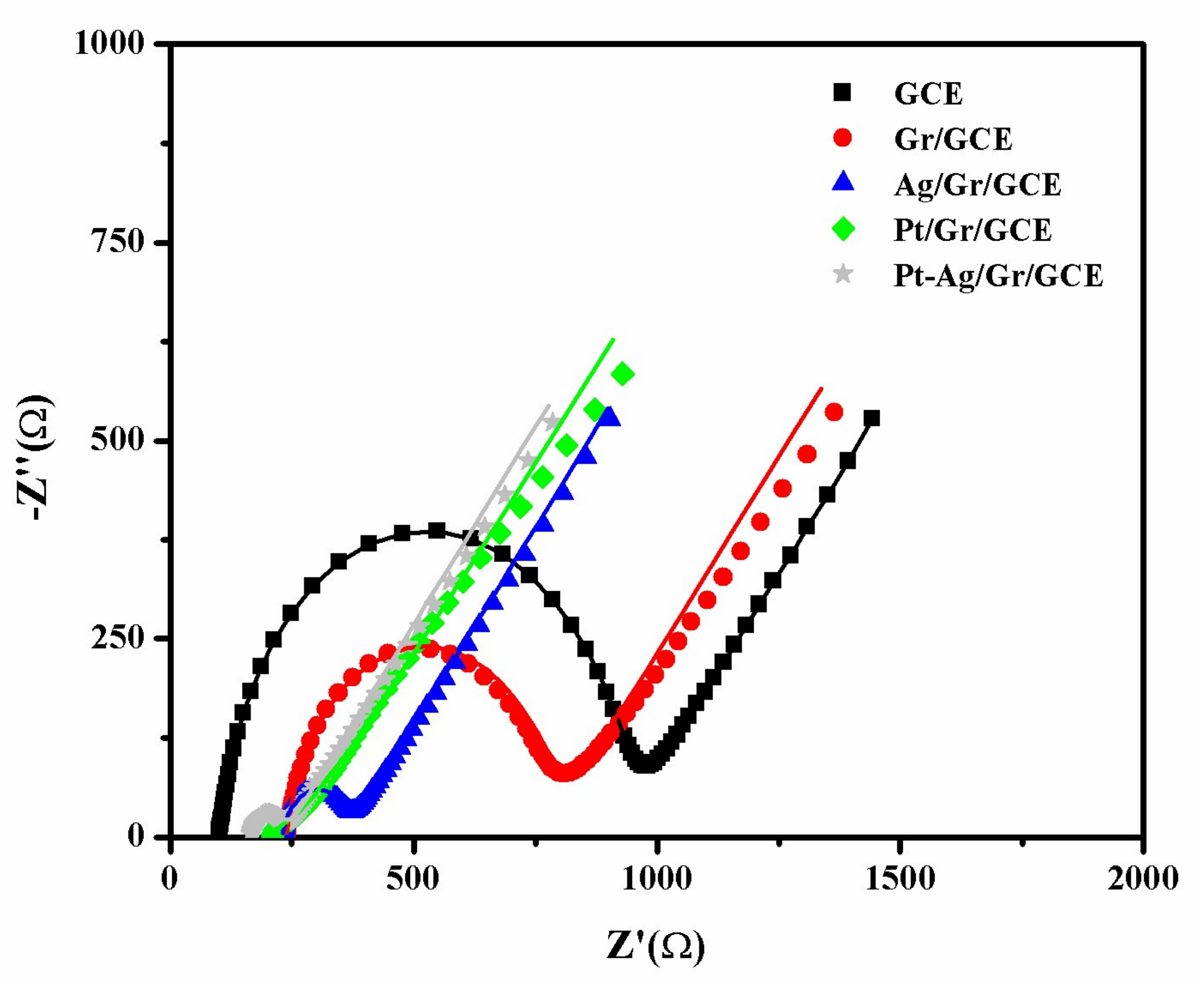

Fig. S3. Nyquist plots of (a) bare GCE, (b) Gr/GCE (c) Ag/Gr/GCE, (d) Pt/Gr/GCE, (e) Pt-Ag/Gr/GCE in 0.1 M KCl with 5.0 mM Fe $[(CN)_6]^{3-/4-}$.

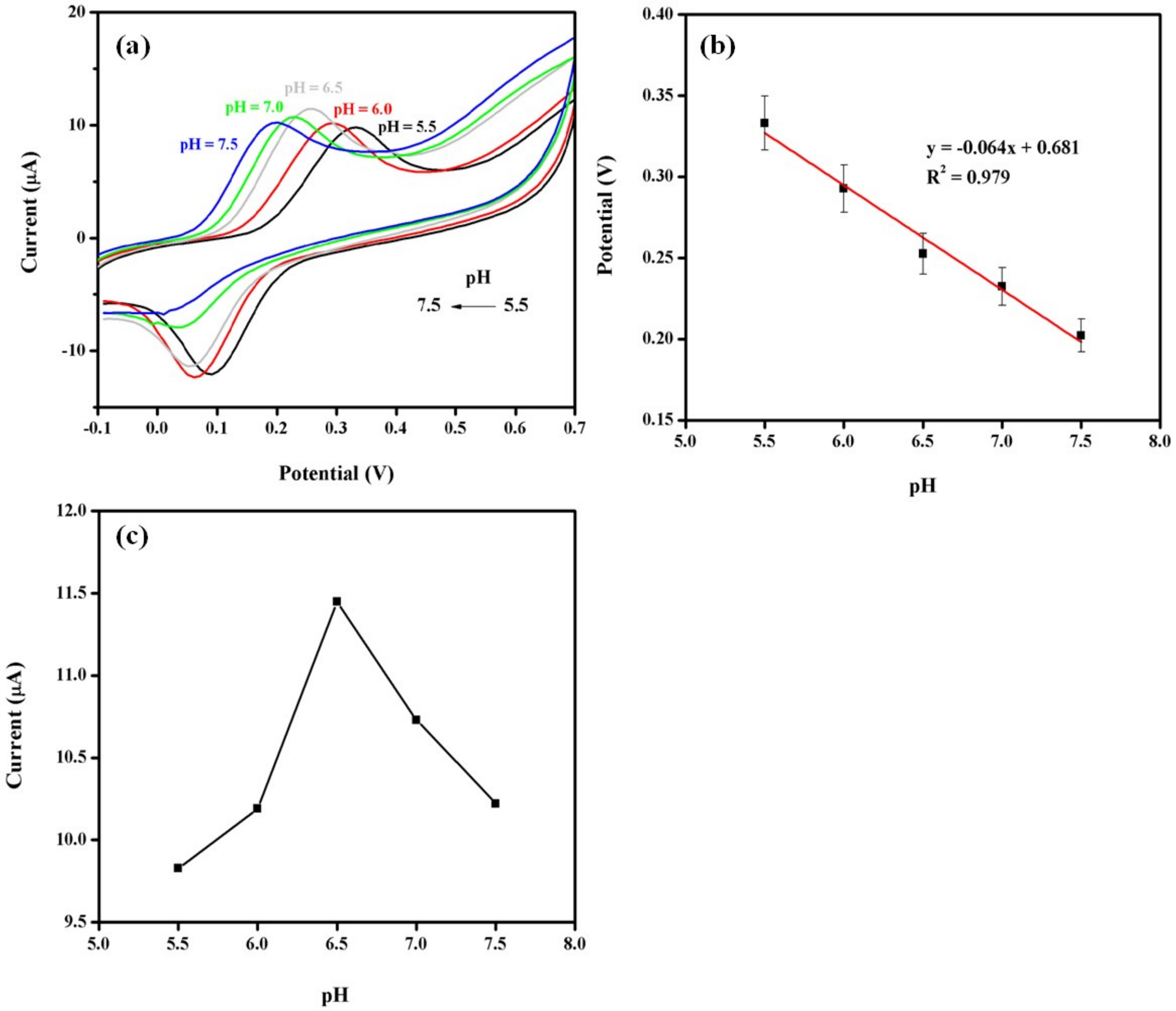

Fig. S4. (a) Cyclic voltammograms of 25 μM DA on Pt-Ag/Gr/GCE at various pH values: 5.5, 6.0, 6.5, 7.0 and 7.5. (b) The dependence of oxidation peak potential on pH values. (c) The dependence of oxidation peak current on pH values.

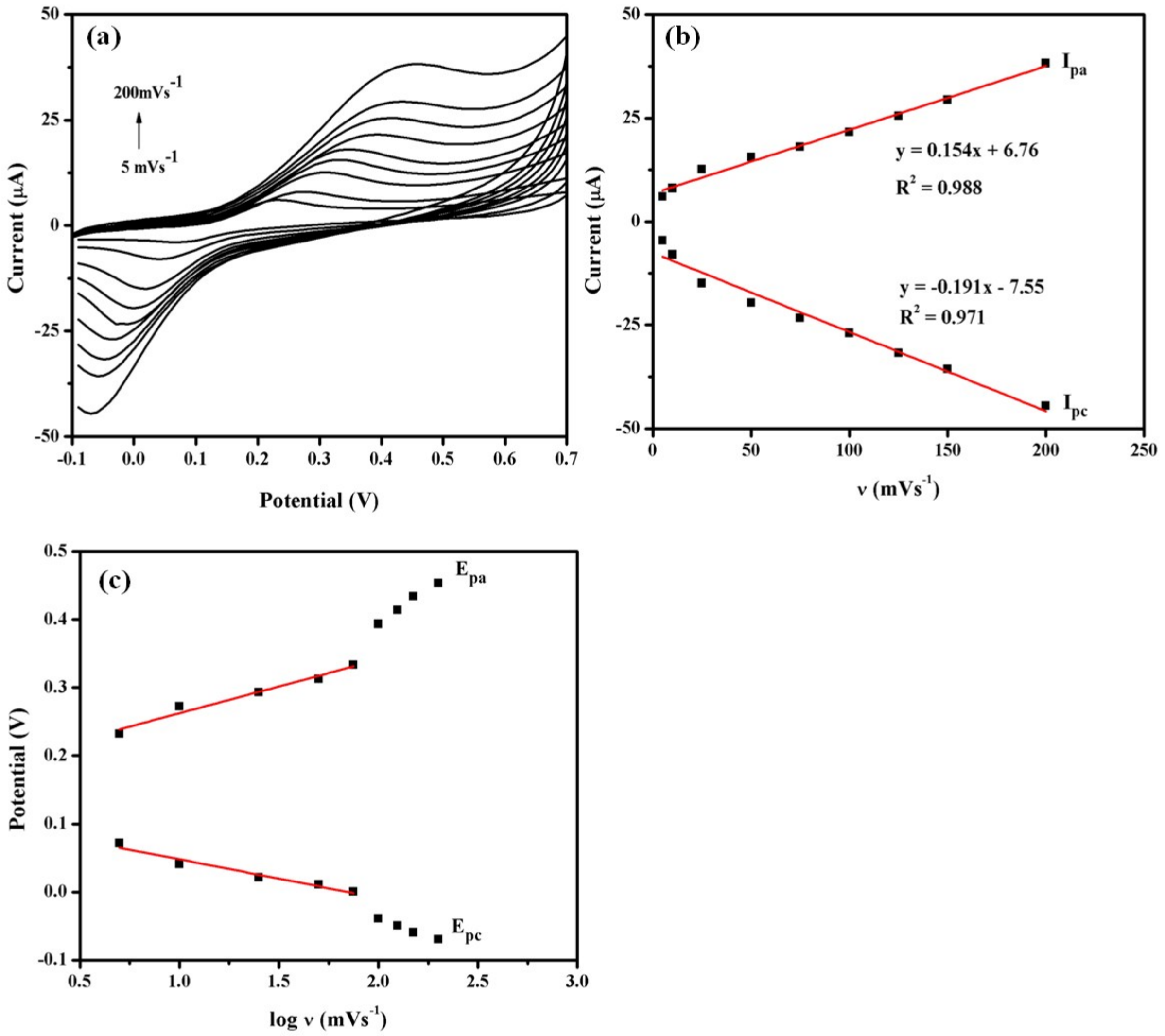

Fig. S5. (a) Cyclic voltammograms of Pt-Ag/Gr/GCE in 25 μM DA at scan rates from 5 to 200 mV s^{-1}. (b) Variation of the redox peak current and scan rate. (c) Variation of the redox peak potential and log of scan rate.

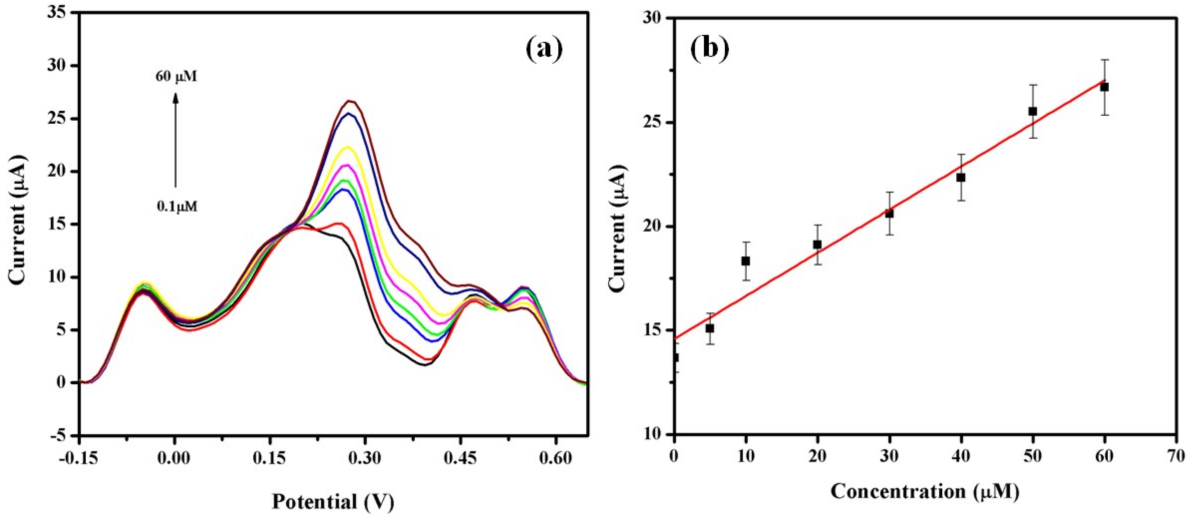

Fig. S6. (a) Differential pulse voltammograms of 1.0 mM AA, 100μM PAP, 100 μM AC, 100 μM UA and different DA concentrations (0.1, 5, 10, 20, 30, 40, 50, 60 μM) at Pt-Ag/Gr/GCE in 0.1 mol L^{-1} PBS at pH 6.5. (b) Relationship of peak current and DA concentration.
